# Emerging Adult Resilience to the Early Stages of the COVID-Pandemic: A Systematic Scoping Review

**DOI:** 10.1007/s10578-023-01585-y

**Published:** 2023-09-12

**Authors:** L. C. Theron, K. Cockcroft, N. Annalakshmi, J. G. Rodgers, T. E. Akinduyo, A. Fouché

**Affiliations:** 1 Department of Educational Psychology, University of Pretoria, Pretoria, South Africa; 2 Department of Psychology, School of Human and Community Development, University of the Witwatersrand, Johannesburg, South Africa; 3 Psychology Department, Bharathiar University, Coimbatore, India; 4 Department of Psychology, School of Health Sciences, North-West University, Vanderbijlpark, South Africa; 5 Department of Social Wellbeing, United Arab Emirates University, Al Ain, United Arab Emirates; 6 Faculty of Education, University of Pretoria, Groenkloof Campus, Pretoria, 0002 South Africa

**Keywords:** COVID-related stressors, Evidence synthesis, Multisystemic resilience, Young adults

## Abstract

Human resilience to COVID-19 related stressors remains a pressing concern following the aftereffects of the pandemic and in the face of probable future pandemics. In response, we systematically scoped the available literature (*n* = 2030 records) to determine the nature and extent of research on emerging adults’ adaptive responses to COVID-19 stressors in the early stages of the pandemic. Using a multisystem resilience framework, our narrative review of 48 eligible studies unpacks the personal, relational, institutional and/or physical ecological resources that enabled positive emerging adult outcomes to COVID-18 stressors. We found that there is a geographical bias in studies on this topic, with majority world contexts poorly represented. Resources leading to positive outcomes foregrounded psychological and social support, while institutional and ecological supports were seldom mentioned. Multisystemic combinations of resources were rarely considered. This knowledge has valuable implications for understanding resilience in the context of other large-scale adverse conditions.

## Introduction

Researcher attention to human resilience, or the capacity for positive outcomes (e.g., mental health) despite exposure to significant stress, continues to surge [1]. The Coronavirus Disease 2019 (COVID-19) pandemic, which was announced by the World Health Organisation on 11 March, is implicit in this surge. Widespread pandemic-related threats to health and wellbeing animated calls to explain, and enable, human resilience to COVID-19 stressors [2]. While pandemic-related stressors prompted creative and innovative responses from some emerging adults (i.e., young people aged 18–29,[3], many faced significant risks to their physical and mental health [4]. Consequently, emerging adult resilience to pandemic-related stressors was labelled a particularly pressing agenda [5]. This scoping review interrogates researcher response to the latter.

The developmental phase of emerging adulthood is associated with specific tasks, including further education or training, career establishment, commitment to a long-term partner, and functional independence [3]. Failure to complete emerging adult developmental tasks results in immediate psychological distress and potentiates long-term negative impacts [6]. Accordingly, it is important to understand, and promote, emerging adult resilience to risks to developmental task fulfilment [7], including their resilience to COVID-19 stressors [5]. Nevertheless, previous reviews and meta-analyses have been inattentive to emerging adult resilience to COVID-19 stressors. They have instead foregrounded COVID-19 risks for emerging adult development, with emphasis on mental illness outcomes [8–13] and vaccination hesitancy [14].

In contrast to these risk-focused reviews, the current review considers the scope (i.e., extent, nature) of studies that investigated emerging adult resilience to COVID-19 stressors. In particular, it seeks to understand how emerging adult resilience was typically explained (i.e., which protective factors were associated with emerging adults’ positive outcomes). This intention is informed by a multisystemic resilience framework – i.e., the understanding that a composite of personal (biological or psychological), relational, institutional, and/or physical ecological (built and natural environment) resources enables positive outcomes in the face of significant stress [1, 15]. For instance, a pre-COVID study of emerging adult resilience to the challenges of structural violence in a South African context reported a combination of protective resources that included personal strengths (i.e., physical health; future-oriented agency), relational resources (i.e., caring family; supportive peers; enabling community), and built environment resources (i.e., an accessible recreation centre) [17].

Although the worst of the COVID pandemic appears to be over globally, the multisystemic sources of emerging adult resilience to COVID-19-related adverse conditions are incompletely understood. They need further investigation and documentation to contribute to the global knowledge base, not least because future pandemics are forecast [18]. While this information will be important for the wellbeing of all young people, its value is heightened for those living in majority world contexts [19]. Following Punch and Tisdall [20], we prefer ‘majority world’ and ‘minority world’ to the more conventional references to ‘the third world’/’Global South’ and ‘the first world’/’Global North’. The term ‘majority world’ signals that “the ‘majority’ of population, poverty, land mass and lifestyles is located in the former, in Africa, Asia and Latin America” [20], p. 241). In using this term, we nudge attention to the resilience of most of the world’s youth (i.e., vast numbers of young people who are typically over-exposed to chronic stress and under-represented in the literature).

Despite growing research on resources that supported young adults during COVID-19 times, no evidence synthesis has been done on these studies. A preliminary search in MEDLINE, the Cochrane Database of Systematic Reviews, and the JBI Evidence Synthesis found no scoping or other systematic reviews with a multisystemic resilience focus on the topic. While there are multiple forms of evidence syntheses, we chose to conduct a scoping review. Scoping reviews aim to synthesise the literature to provide a broad overview of a specific topic, provide insight into how that topic has been researched, and inform future scientific inquiry [21]. Our review question, and inclusion/exclusion criteria were developed using the Joanna Briggs Institute (JBI) PCC (Participant; Concept; Context) framework [21]. The following broad question informed our scoping review: what resources supported the resilience of emerging adults (as evidenced in positive outcomes) during the early stages (i.e., January 2020 to June 2021) of the COVID-19 pandemic? Following vaccination rollout to the public (typically toward the end of 2020 in minority world contexts like North America and Europe and around mid-2021 in majority world countries; [22, 23], COVID-related distress was less pronounced than in earlier pandemic stages [24]. Earlier stages were generally characterised by lockdown-related disruptions to daily routine, education, livelihoods, and relationships, as well as significant contagion/mortality fears. Understanding what supported resilience to these stressors will advance pro-active preparation for future pandemics [18], with emphasis on championing resilience from the earliest stages of future pandemics.

## Method

To conduct the scoping review, we followed the steps originally advised by Arksey and O'Malley [25] and then others [21, 26, 27]. Reporting of the findings aligns with the PRISMA extension for Scoping Reviews (PRISMA-ScR) [27].

### Eligibility Criteria

To be included, papers needed to report (i) an empirical study (quantitative, qualitative, or mixed methods) that (ii) investigated any positive outcome among emerging adults (i.e., 18–29-year-olds; [3] exposed to COVID-19 related stress and (iii) the protective factor/s associated with those positive outcomes. In line with the resilience literature [28], a positive outcome in the face of COVID-related stressors could include physical health, mental health (e.g., limited/no symptoms of depression), subjective wellbeing, quality of life, engagement in education, and/or academic progress/achievement. However, we excluded studies that reported interventions to support these outcomes or that recommended/theorised how to achieve positive outcomes during COVID-challenged times. We excluded studies that implied that participants could be emerging adults (e.g., references to students), but that provided no proof (i.e., average age or age range consistent with emerging adulthood). We also excluded studies that had a range of participants (e.g., adolescents to elderly persons), but reported no findings that were specific to emerging adults.

### Information Sources and Search

A trained research assistant (i.e., a research psychology Master’s student) searched for relevant academic journal publications using multiple databases: Africa-Wide, CINAHL, ERIC, PsycARTICLES, PsycINFO (all via EBSCOhost platform); Medline (via Web of Science Clarivate Analytics); PubMed; Scopus (which includes contents of Embase); Web of Science Core Collection; and SciELO Citation Index. Given concerns about the quality of many COVID-19-related studies [29], we delimited eligibility to full-text journal papers. The search was conducted in September 2021 to retrieve eligible studies published between January 2020 and August 2021.

Because of extensive prior experience in conducting resilience-focused reviews [16, 30–35], we did not invite a librarian to draft the search strategy. We repeated the search terms from those reviews and added COVID-19. The search terms were: Resilien* or strengths or coping or hardiness or adaptation or grit or perseverance or protective factors or promotive factors or buffer* or positive adjustment or positive effects or benefits AND emerging adult or college student or young adult or early career or young people or youth AND COVID-19 or Coronavirus or 2019-ncov or sars-cov-2 or cov-19 or covid pandemic. The search terms were applied to titles, abstracts, and keywords/subject terms.

**Fig. 1:**
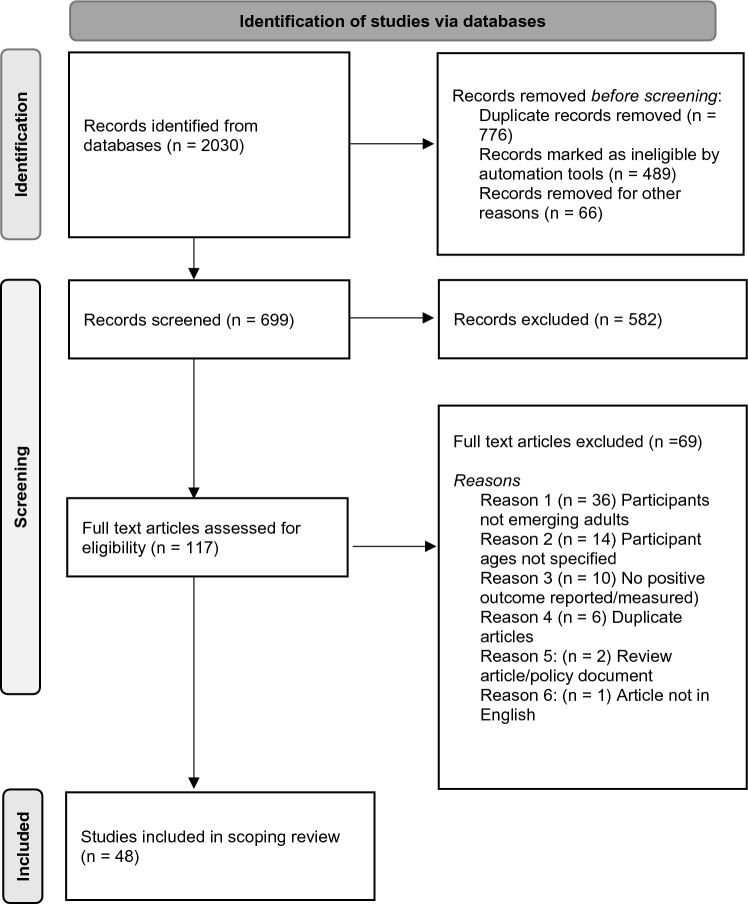
Prisma Diagram

In total, the search yielded 2030 records (see Fig. 1). We exported a detailed view of each record into Endnote. We used this software to identify duplicates and ineligible records (*n* = 1331; see Fig. 1). Before deleting the duplicates, we verified that the record was in fact a duplicate. The removal of these records resulted in 699 records for screening.

Two authors (AF; LT) screened the records. Using the blind procedure function in Rayyan software, they independently perused the titles and abstracts of all records to confirm record consistency with specified eligibility criteria (i.e., empirical study documenting positive emerging adult outcomes during COVID-19-challenged times and the protective factors associated with those outcomes). Following Saldana [36], they held consensus discussions to resolve the isolated discrepancies (*n* = 29; 4.1% of the records). The screening resulted in 117 records that were considered for selection. After a decision was made on the included studies, the reference lists of the included studies were screened to identify further eligible studies. No further eligible studies were found.

### Selection of Sources of Evidence

A post-doctoral fellow (TA) and qualified research psychologist (GR) independently read the full texts to confirm their fit with the specified eligibility criteria. Following a consensus discussion to resolve the discrepancies in their assessments (*n* = 8; 6.8% of the records), they recommended exclusion of 69 full texts (see Fig. 1). Two authors (AN; KC) confirmed their recommendations.

### Data Charting Process

To chart the data, AF and LT designed a data charting form. It included the study’s purpose; date/s when study conducted; geographical context; design; sample (size and specifics); positive outcomes and how they were measured/investigated; and factors that were associated with positive outcomes. Once they had piloted the chart with 10 full texts, they shared it with TA and GR who independently extracted data from the remaining full texts. AF, AN, KC, and LT confirmed and, where necessary, refined that data extraction.

### Collating, Summarising and Reporting the Results

Guided by Petticrew and Roberts [37], and JBI's recent publication on qualitative content analysis in scoping reviews [38], we conducted a narrative synthesis. We tabulated essential aspects of the included studies, including their design, location, and positive outcome/s reported. We were particularly attentive to the protective factors associated with these outcomes. In line with the multisystemic resilience framework [1, 15], we considered the nature of these protective factors and how often that nature reflected a combination of factors (e.g., psychological strengths and social supports) versus single system factors (e.g., only psychological strengths).

## Results

Our search generated 2030 records, of which 699 were screened after removal of duplicates and records marked as ineligible by automation tools. Of these, 117 full text articles were retrieved and assessed for eligibility, and 48 were subsequently included in the review (see Fig. 1). We summarise key details of the included studies in Table 1. In what follows, we provide an overview of these studies (i.e., where conducted, design detail, and positive outcomes of interest), before detailing patterns in the protective factors associated with the positive outcomes that the studies reported.

**Table 1:** Summary of positive outcomes reported in eligible studies and protective factors associated with them

Studies reporting protective factors from two or more systems					
Authors	Location	Study design & implementation date	Sample	Positive outcome	Multisystemic protective factor combination associated with positive outcome
Agbaria & Mokh [74]	Israel	Cross-sectional survey First 3 months of C19 outbreak in Israel, 2020	*N* = 625 (72% female) Israeli-Palestinian college students Age range: 19–30 Mage = 24.8 (SD = 5.88)	Coping with stress	Problem-focused coping style; adaptive personality traits; social support
Germani, Buratta, Delvecchia, Gizzi, & Mazzeschi [67]	Italy	Cross-sectional survey 17–26 March 2020	*N* = 1045 (70% female) Age range: 18–29 Mage = 24.18 (SD = 3.60)	Less severe anxiety	Interpersonal functioning (secure attitude in relationships); personal functioning (self-esteem, self-efficacy); general health status
Gittings et al. [48]	South Africa	Cross-sectional qualitative interview study April 2020	*N* = 12 (50% female) Age range: 18–25 Mage = –	Well informed about C19 prevention	Knowledge/enactment of C19 mitigation measures; effective public health campaign
Golemis et al. [86]	Greece	Cross-sectional survey 10–13 April 2020	*N* = 1559 ( 3.2% female) 50.7% tertiary students Age range: 18–30 Mage = –	Reduced loneliness; higher social responsibility	Social interaction (sharing thoughts and feelings about COVID-19); practicing sports; humour; religious activity
Hou et al. [75]	China	Cross-sectional survey March 2020	*N* = 1251 (62.2% female) College students Age range: 18–25 Mage = 20.92 (SD = 1.47)	Reduced depression	Social support; grit
Hu & Morrison Gutman [84]	UK	Longitudinal study June–November 2020	*N* = 419 (70.6% female) Age range: 18–25 Mage = –	Reduced loneliness	Employed/engaged in education; higher household income; emotional support (males only)
Hyun et al. [42]	USA	Cohort study Wave (W) 1: April – August 2020 W2: September 2020 – March 2021	*N* = 805 (completed W1 & W2; (84.8% female) 56.4% students Age range: 18–30 Mage = 24.8 (SD = 3.30)	Post-traumatic growth	Psychological resilience; family connectedness;
Jin et al. [76]	China	Cross-sectional survey March 2020	*N* = 847 (77.6% female) Undergraduate students Age range: – Mage = 20.09 (SD = 1.168)	Less problematic cellphone use	Personal resilience; sense of school belonging
Lardone et al. [77]	Italy	Cross-sectional survey 27 April–11 May 2020	*N* = 213(57.7% female) 83.6% students Age range: – Mage = 21.2 (SD = 3.5)	Quality of life	Social connectedness; C19 fears and support for public health; C19 fears and social identity affirming behaviours
Li, Liu et al. [79]	China	Cross-sectional survey 3–15 March 2020	*N* = 1676 (64.9% female) Undergraduate students Age range: – Mage = 20.17 (SD = 1.497)	Lower health anxiety	Higher C19-related knowledge (understanding of risks; knowledge of preventive behaviours); media-facilitated information and trust in mainstream media
Li & Peng [78]	China	Cross-sectional survey 21 – 24 February 2020	*N* = 2640 (68.79% female) 85.49% undergraduate students 14.51% graduate students Age range: 18–25 Mage = –	Reduced anxiety	Coping strategies; social support (family support; counsellor support)
Liu et al. [83]	USA	Cross-sectional survey 13 April – 19 May 2020	*N* = 898 (81.3% female) 61.3% students Age range: 18–30 Mage = 24.5	Below clinical levels of anxiety, depression, PTSD	Personal resilience; family support; instrumental support
Marchini et al. [80]	Belgium; Italy	Cross-sectional survey 7 April – 4 May 2020	*N* = 825 (74% female) 70.9% tertiary students Age range: 18–25 Mage = 22	Mental health (operationalised as never needing to seek mental health support)	Personal resilience; social supports
Nola et al. [81]	Italy	Cross-sectional pilot survey 20 June – 20 July 2020	*N* = 174 (69.5% female) University students Age range: – Mage = 21.70 (SD = 2.15)	Reduced anxiety	Personal interests; medium—high quality relationships
Nomura et al. [82]	Japan	Cross-sectional survey 20 May – 16 June 2020	*N* = 2712 (46.2% female) University students Age range: – Mage = 20.5 (SD = 3.5)	Reduced depression; reduced suicide ideation	Exercise; someone to consult about worries
Oswald et al. [85]	Australia	Cross-sectional survey 17 November 2020 – 9 January 2021	*N* = 1004 (55% female) 66% students Age range: 18–24 Mage = 21.23 (SD = 1.93)	Flourishing mental health	Secure employment; using screen time to connect with others; high levels of hope; incidental and purposive contact with nature
Raj & Bajaj [49]	India	Cross-sectional phenomenological study March 2020	*N* = 12 (58% female) Age range: 26–30 Mage = –	Coping with living alone during lockdown	Constructive meaning-making; health-promoting behaviours; social support/enabling human interaction
Son et al. [51]	USA	Mixed methods study May 2020	*N* = 195 (57% female) Undergraduate students Age range: – Mage = 20.7 (SD = 1.7)	Stress and anxiety management	Coping strategies; social support (family; friends)
Suhail et al. [50]	India	Cross-sectional phenomenological study March 2020	*N* = 10 (80% female) College students Age range: 20–27 Mage = 23.8	Management of C19 stress	Observing preventive measures; health-promoting lifestyle changes; recreational activity; contact with friends (via social media)

Studies reporting protective factors from a single system [i.e., the self]					
Authors	Location	Study design & implementation date	Sample	Positive outcome	Personal protective factors associated with positive outcome
Allesandri et al. [69]	Italy	Cross-sectional survey 1—15 March 2020	*N* = 287 (52% female) Age range: – Mage = 22.68 (SD = 2.62)	Lower levels of daily negative affect	Self-concept clarity
Alsolais et al. [55]	Saudi Arabia	Cross-sectional survey 22 April —16 May 2020	*N* = 492 (55.7% female) Nursing students Age range: – Mage = 21.77 (SD = 2.47)	Reduced depression, stress, and anxiety	Active coping strategies (including positive reframing); substance use
Dongmei [56]	China	Cross-sectional survey March 2020	*N* = 600 (46% female) Age range: 18–22 Mage = –	Reduced social anxiety	Psychological capital; coping skills
Eden et al. [57]	USA	Cross-sectional survey 23 March – 17 April 2020	*N* = 425 (68.5% female) University students Age range: – Mage = 20.19 (SD = 2.18)	Psychological wellbeing	Traits (resilience, optimism, hope); media-based coping strategies
Germani, Buratta, Delvecchia, & Mazzeschi [68]	Italy	Cross-sectional survey 17 – 26 March 2020	*N* = 1011 (71.2% female) Age range: 18–29 Mage = 24 (SD = 3.60)	Lower psychological maladjustment	Collectivistic orientation
Guan et al. [40]	China	Cross-sectional survey 4—12 February 2020	*N* = 24,678 (44.8% female) College students Age range: 18–25 Mage = 20.51 (SD = 1.28)	Reduced anxiety	High C19 knowledge; C19 mitigation behaviour (e.g., mask wearing); demographic factors (male; rural residence)
Khalid et al. [66]	Pakistan	Cross-sectional survey February – May 2020	*N* = 937 (39.4% female) Under- & post-graduate college/university students Age range: ≤ 20 – ≥ 26 Mage = 22 (SD = 3.01)	Reduced psychological distress	High C19 knowledge
Kornilaki [58]	Greece	Cross-sectional survey 7 – 14 April 2020	*N* = 1018 (83% female) Undergraduate students Age range: – Mage = 21.5 (SD = 4.2)	Reduced psychological distress; less negative affect	Daily routine; altruism
Labrague & Ballad [59]	Phillipines	Cross-sectional survey 6^th^ month of mandatory lockdown 2020	*N* = 243 (81.5% female) Undergraduate college students Age range: – Mage = 20.77 (SD = 2.66)	Reduced lockdown fatigue	Personal resilience; coping skills; demographic factors (male)
Li, Xu, He et al. [70]	China	Cross-sectional survey 2—12 June 2020	*N* = 424 (72.6% female) College students Age range: – Mage = 20.49 (SD = 1.95)	Reduced psychological distress	Sense of coherence
Lin [71]	China	Prospective survey study Wave (W) 1: 27 December 2019 – 1 January 2020 W2: 15 February – 14 March 2020	College students *N*_*W1*_ = 319 (51.72% female) Age range: – Mage = 20.30 (SD = 1.46) *N*_*W2*_ = 154 (69.48% female) Age range: – Mage = 20.41 (SD = 1.45)	Life satisfaction; reduced psychological distress; C19 mitigation behaviour; prosocial behaviour	Meaning in life
Mushquash & Grassia [39]	Canada	Cross-sectional survey May 2020	*N* = 131 (80.9% female) College students Age range: – Mage = 20.32 (SD = 2.70)	Reduced psychological distress	Adaptive engagement coping
Padmanabhanunni & Pretorious [63]	South Africa	Cross-sectional survey March –June 2020	*N* = 337 (77.2% female) University students Age range: – Mage = 21.95 (*SD* = 4.7)	Less loneliness	Personal resilience
Padmanabhanunni & Pretorious [62]	South Africa	Cross-sectional survey March –June 2020	*N* = 337 (77.2% female) University students Age range: – Mage = 21.95 (*SD* = 4.7)	Less loneliness	Ego resilience; life satisfaction
Savitsky et al. [60]	Israel	Cross-sectional survey 3^rd^ week of lockdown 2020	*N* = 215 (88% female) Nursing students Mage^1st year^ = 23.4 (SD = 2.8) Mage^2nd year^ = 25.1 (SD = 2.3) Mage^3rd year^ = 26.1 (SD = 3.0) Mage^4th year^ = 27.9 (SD = 3.4)	Reduced anxiety	Personal resilience; coping strategy: humour
Shanahan et al. [44]	Switzerland	Prospective-longitudinal cohort study April 2020	*N* = 786 (48.1% female) Age: 22	Reduced distress	Coping strategies (daily routine, physical activity, positive reappraisal/reframing, acceptance, maintaining contact with family/friends)
Sweeny et al. [72]	China	Cross-sectional survey 12—19 February 2020	*N* = 5115 (72.8% female) Age range: – Mage = 21.36 (SD = 4.39)	Psychological wellbeing	Flow; mindfulness
Tan et al. [64]	China	Cross-sectional survey September—October 2020	*N* = 1871 (67% female) College students Age range: – Mage = 20.6 (SD = 1.0)	Psychological wellbeing	Personal resilience
Vidas et al. [61]	Australia	Cross-sectional survey April—June 2020	*N* = 402 (73% female) University students Age range: – Domestic student Mage = 19.1 (SD = 4.10) International student Mage = 20.1 (SD = 5.46)	Psychological wellbeing	Coping strategy (listening to music)
Xia et al. [73]	China	Cross-sectional survey 1 July – 1 September 2020	*N* = 494 (71.5% female) Residential college students Age range: – Mage = 19.69 (SD = 1.327)	Lower probability of problematic internet use	Internal locus of control
Ye et al. [65]	China	Cross-sectional survey Winter recess, 2020	*N* = 1293 (52% female) College students Age range: – Mage = 20.79 (SD = 1.67)	Reduced depression	Personal resilience
Zhang, Lin, et al. [45]	China	Cohort study Time (T) 1: 9 – 15 February 2020 T2: 16 – 26 April 26, 2020	Medical students *N*_*T1*_ = 1069 (58.65% female) Mage = 20.93 (SD = 1.64) *N*_*T2*_ = 1511 (53.01% female) Mage = 21.21 (SD = 1.69)	Reduced depression/anxiety	Personal action (more frequent handwashing)
Zhang, Wang, et al. [46]	China	Cross-sectional survey Daily, 23 – 29 March 2020	*N* = 391 (63.4% female) College students Age range: – Mage = 20.77 (SD = 1.01)	Positive affect	Personal resilience (especially optimism); male sex

Studies reporting protective factors from a single system [i.e., social system]					
Authors	Location	Study design & implementation date	Sample	Positive outcome	Protective relational factors associated with positive outcome
Li, Wu, Meng et al. [52]	China	Cross-sectional survey 2–15 March 2020	*N* = 450 (61.9% female) College students Age range: 18–22 Mage = 19.1 (SD = 0.92)	Psychological adjustment (i.e., fewer self-reported symptoms of anxiety, depression, PTSD)	Social support
Pompili et al. [53]	Italy	Cross-sectional survey April–May 2020	*N* = 447 (62.63% female) 62% students Age range: 18–26 Mage = 23 (SD = 1.93)	Less disordered eating and alcohol consumption	Living with family; social support
Porter et al. [43]	Ethiopia, India, Peru & Vietnam	Cohort study August–October 2020	*N* = 8988 (49% female) Age range: 18–19; 25—26 Mage = –	Better mental health (i.e., less anxiety/depression)	Prior parent/peer relationships
Woznicki et al. [54]	USA	Cross-sectional survey July 2020	*N* = 183 (63.4% cisgender female) LGBQ sample Age range: 18–23 Mage = 20.19 (SD = 1.58)	Reduced depression	Family support; when family support low: parasocial (technology-facilitated) relationships
Xu et al. [47]	USA	Cross-sectional phenomenological study	*N* = 14 (92.8% female) 85% Chinese international college students Age range: 18–22 Mage = 30 (SD = 0.92)	Management of C19 stressors	Supportive professors and peers

Studies reporting protective factors from a single system [i.e., the macrosystem]					
Authors	Location	Study design & implementation date	Sample	Positive outcome	Macro-level protective factors associated with positive outcome
Bleil et al. [41]	USA	Prospective cohort study June–August 2020	*N* = 374 (57% female) Age range @time of follow-up: 28.6–29.5 Mage = 29.1 (SD = 0.2)	Positive change in response to C19	Early life (i.e., childhood) exposure to neighbour hood adversity

### Overview of the Included Studies

Nineteen countries were represented in the included studies (i.e., Australia; Belgium; Canada; China; Ethiopia; Greece; India; Israel; Italy; Japan; Pakistan; Peru; the Philippines; Saudi Arabia; South Africa; Switzerland; the UK; the USA; Vietnam). Most studies were conducted in Asia and the Pacific (*n* = 23), with China being most prominent within that region (*n* = 15). Europe (*n* = 10), with emphasis on Italy (*n* = 7), was fairly well represented, as was North America (*n* = 8). Three studies were conducted in the Middle East, three in sub-Saharan Africa, and one in South America.

Most included studies employed a cross-sectional survey design (*n* = 36; see Table 1). These studies typically sampled college/university students in which young women represented the majority; sample sizes ranged from *N* = 131 [39] to *N* = 24,678 [40]. Longitudinal studies were scarce (*n* = 5), and in all instances relied on cohorts that were established prior to the COVID-19 pandemic [41–45]. Qualitative studies were also rare (*n* = 4), and except for Xu et al. [47], conducted in majority world countries (i.e., South Africa, [48], India, [49, 50]. Only one of the included studies, i.e., Son et al.’s [51] study with undergraduates in the USA, reported a mixed methods design.

In most included studies, the positive outcome of interest related to mental health (typically lower levels of psychological distress, including anxiety and depression). A handful of included studies (*n* = 6) focused on constructive management/avoidance of loneliness and/or lockdown fatigue. Only five of the included studies reported positive psychology outcomes (i.e., flourishing, quality of life; post-traumatic growth/positive change; life satisfaction). A single study reported knowing how best to avoid/limit COVID-19 contagion as a positive outcome [48].

### Patterns in the Protective Factors Associated with Emerging Adult Resilience to COVID-19 Stress

As summarised in Table 1, the included studies reported a variety of protective factors associated with positive outcomes in the face of COVID-19-related challenges, including personal resources (e.g., constructive coping skills or an altruistic disposition), relational resources (e.g., supportive family or friends), and less often, institutional or ecological resources (e.g., employment; access to sports facilities or green spaces). Closer inspection of these protective factors showed two prominent patterns: inattention to multisystemic resilience resource combinations (i.e., personal resources dominated accounts of emerging adult resilience to COVID-19 stressors) and, when resource combinations were reported, psychological resources and social supports were preponderant. These patterns are detailed next.

#### Inattention to Multisystemic Resource Combinations

Most included studies (*n* = 29) did not report a combination of resources that were distributed across multiple systems (e.g., the self, family, and built environment). Instead, and as explained below, they typically reported only personal resources (*n* = 23). A few (*n* = 5) reported only relational resources (i.e., family, peers, and/or supportive professors) and associated social support, [43, 47, 52–54]. A single study reported neighbourhood factors only (i.e., childhood exposure to neighbourhood disadvantage) and theorised how this exposure facilitated psychological steeling that scaffolded positive responses to COVID-related challenges [41].

In the studies reporting personal strengths only, adaptive coping skills (e.g., hopeful meaning making, listening to music, judicious media use, or maintaining a daily routine) were prominently associated with positive outcomes (*n* = 9; [39, 44, 55–61]). Personal or psychological resilience was reported almost as regularly as coping skills (*n* = 8), but variably operationalised. Operationalisations included psychological capital [56], ego resilience [62], capacity to ‘bounce back’ [59, 63], and/or assets/resources at the level of the individual [46, 60, 64, 65].

Less commonly reported personal protective factors included being well informed about/enacting COVID-19 mitigation measures [40, 45, 66]. Similarly, only two studies associated young people’s altruistic or collectivist orientation with positive outcomes; both were conducted with young people from European countries that value family and community and encourage collectivist values [58, 68]. There was isolated consideration of the protective value of traditional and/or positive psychology constructs, including self-concept clarity [69]; sense of coherence Li, Xu, He et al., [70]; meaning in life [71], life satisfaction [62], mindfulness and flow [72], and internal locus of control [73].

#### Resource Combinations Foreground Psychological Resources and Social Supports

Nineteen studies associated multiple resources with emerging adult positive outcomes in the face of COVID-related stressors. Most (*n* = 13) reported a combination that drew on personal and relational factors [42, 49, 51, 67, 74–78, 80, 81, 83]. While these studies seldom specified details of the relational resources (e.g., they referred broadly to social support), four did specify family [42, 51, 78, 83], one referred to friends [51], and one included mental health practitioners [78]. Only two studies (i.e., [67, 81] specified that young people needed to experience the relationships in question as secure/having medium to high quality for them to be protective.

Resource combinations seldom reported institutional supports. Exceptions included reference to effective public health campaigns [48], opportunity for employment and/or education [84, 85], and media-facilitated information that was trustworthy [79]. Similarly, combinations rarely included resources in the physical ecology. The only study to explicitly report physical ecological resources was Oswald et al. [85]. In addition to secure employment, social interaction and hopefulness, this Australian study associated unintentional or intentional contact with nature (e.g., outdoor garden) with young people’s capacity to flourish. Two other studies implied physical ecological resources in the resource combinations they reported. While detailing ways that Indian young adults coped adaptively with COVID-related stressors, Suhail et al. [50] reported a participant’s account of taking their dog for a walk and of appreciating nature. Similarly, Golemis et al. [86] included sporting and religious activity in the resource combination that protected Greek participants thereby suggesting access to outdoor/indoor spaces that facilitated sporting activity.

## Discussion

Our aim with this paper was to scope the literature to determine the nature and extent of researcher response to calls to account for human resilience—especially emerging adult resilience—to COVID-19 stressors [2, 5]. In particular, we were interested in understanding how emerging adult resilience was typically accounted for during the early stages of the pandemic (i.e., patterns in the protective factors associated with emerging adults’ positive outcomes). We believe that these insights are pivotal to emerging adult resilience to subsequent pandemics, but also to how researchers conceptualise future emerging adult resilience studies.

Our review points to a geographical bias in studies of emerging adult resilience to COVID-19 stress. While it is heartening that researcher response to calls to investigate emerging adult resilience included young people from multiple countries across multiple regions, studies in Asia and the Pacific (especially China) were predominant, followed by North America (especially the USA) and Europe (especially Italy). This apparent bias could relate to the pandemic originating in Asia (specifically China) and/or European countries (including Italy) and the USA reporting of the highest COVID-19 infection rates globally [87]. However, it is also possible that it is a sign of researchers being under-attentive to emerging adults in majority world contexts like Africa and South America. Certainly, this trend was reported in pre-COVID-19 reviews of young people’s mental health (e.g., [16, 88]. Given the relentless challenges that demand resilient responses from young people in majority world contexts like South America and Africa [89], and the concerns that future pandemics will (again) have disproportionately negative impacts on disadvantaged majority world youth [19], it is important that studies of their resilience be fast-tracked.

Our review also identified a sampling bias in studies of emerging adult resilience to COVID-19 stress, in that college/university students dominated the samples. While college and university closures and the introduction of virtual classes meant that students were particularly vulnerable to stress during the early stages of the pandemic [90], the over-representation of students in the studies we reviewed raises questions about the applicability of the findings to emerging adults not involved in employment, education, or training (NEET). Pre-Covid, NEETs were already a growing population in need of intervention [91]. COVID-related disruptions to livelihoods and economies make understanding of resilience among NEET emerging adults even more pressing.

Of most concern, however, is that our review shows that studies of emerging adult adaptation to early pandemic stressors were inclined to perpetuate outdated understandings of resilience as a solo endeavour. More studies reported personal strengths than studies reporting resource combinations. While personal strengths are important resilience-enablers, they cannot fully account for why stress-exposed young people show positive outcomes [28]. Instead, as multisystemic resilience frameworks show, positive outcomes are co-enabled by a combination of resources that go beyond personal strengths [1, 15], also among emerging adults [17, 92].

While it was reassuring to see that 19 studies did not restrict what enabled positive outcomes to personal strengths, most (n = 13) of these studies reported an attenuated combination (i.e., personal and relational resources). If emerging adult resilience is to be optimized, particularly during pandemic-challenged times, then more studies like that of Oswald et al. [85] are urgently needed. Their study was distinguished by its explicit investigation of multiple resources and multiple system levels, including the physical ecology. Future studies of emerging adult resilience need to be purposefully multisystemic. Put differently, they need to be designed to investigate the biological, psychological, relational/social, institutional, and physical ecology resources that matter for emerging adult resilience and to consider how identified resource combinations are responsive to situational and cultural context [15]. Moreover, they need to consider what the optimal number of resources in such a resource combination might be [93]. The latter is particularly important in pandemic-challenged times that are associated with austerity and resource constraints.

## Limitations

Early adaptive responses, like those documented in this review, might differ from those in later stages of the pandemic, especially once vaccines were freely available [94]. A follow-up review would address this limitation. In addition, the focus on student samples limits the generalizability of findings beyond this group. The exclusion of grey literature from the review means that some unpublished studies that present different findings are not reflected here.

## Conclusion

The decline in pandemic related stressors should not breed complacency. Future pandemics are likely [18]. A takeaway from our review of what enabled the resilience of emerging adults during the early stages of the COVID-19 pandemic is that preparation aimed at advancing resilience to future pandemics must shift its focus from individual resources to resource combinations rooted in multiple systems. In doing so, optimal preparation will require special attention to the capacity of education, health, economic and built/natural ecology systems to support emerging adult resilience. Continued inattention to these broader systems will force emerging adults and their families/friends to continue to take primary responsibility for positive adjustment to future stressors and support the longevity of neoliberal agendas.

## Summary

This scoping review provides a detailed overview of the nature and extent of empirical research on emerging adults’ adaptive responses to COVID-19 stressors in the early stages of the pandemic. Following PRISMA guidelines, we included 48 studies of emerging adult resilience to COVID-19 stressors. Using a multisystem resilience framework and narrative review approach, we found that most studies reported person-focused or individualised accounts of young people’s resilience (despite such narrow accounts being disparaged by recent developments in resilience science). Multisystemic combinations of resources were rarely considered (despite new developments in resilience science pointing to the salience of a mix of multisystemic resources to youth resilience). When studies did report a combination of resources, they foregrounded psychological and social supports and seldom mentioned institutional and ecological supports. There was also a geographical bias in the included studies, with the majority world contexts of Africa and Latin America poorly represented. This synthesis advances a multisystemic research agenda informed by resilience studies that are purposefully designed to measure biological, psychological, social, institutional, and environmental resources in order to more fully account for young people’s capacity to respond adaptively to significant stress. Also, these studies should purposefully end the historic researcher neglect of young people in majority world contexts, with special attention to young people in Africa and Latin America.

## Data Availability

Included publications are marked with * in the reference list; the extracted data are included in Table 1.
